# Performance of pulse oximeters as a function of race compared to skin pigmentation: a single center retrospective study

**DOI:** 10.1007/s10877-024-01211-9

**Published:** 2024-08-28

**Authors:** Audrey I. Marlar, Bradley K. Knabe, Yasamin Taghikhan, Richard L. Applegate, Neal W. Fleming

**Affiliations:** 1 Davis School of Medicine, University of California, Sacramento, CA USA; 2 Department of Anesthesiology, Loma Linda University, Loma Linda, CA USA; 3 Department of Anesthesiology & Pain Medicine, Davis School of Medicine, University of California, Sacramento, CA USA; 4 Department of Anesthesiology & Pain Medicine, UC Davis Medical Center, 4150 V Street PSSB – Suite1200, Sacramento, CA 95817-1460 USA

**Keywords:** Massey-Martin scale, Oxygenation, Pulse oximeters, Race, Racial bias, Skin pigmentation

## Abstract

Pulse oximetry (SpO_2_) is a critical monitor for assessing oxygenation status and guiding therapy in critically ill patients. Race has been identified as a potential source of SpO_2_ error, with consequent bias and inequities in healthcare. This study was designed to evaluate the incidence of occult hypoxemia and accuracy of pulse oximetry associated with the Massey-Martin scale and characterize the relationship between Massey scores and self-identified race. This retrospective single institute study utilized the Massey-Martin scale as a quantitative assessment of skin pigmentation. These values were recorded peri-operatively in patients enrolled in unrelated clinical trials. The electronic medical record was utilized to obtain demographics, arterial blood gas values, and time matched SpO_2_ values for each PaO_2_ ≤ 125 mmHg recorded throughout their hospitalizations. Differences between SaO_2_ and SpO_2_ were compared as a function of both Massey score and self-reported race. 4030 paired SaO_2_-SpO_2_ values were available from 579 patients. The average error (SaO_2_-SpO_2_) ± SD was 0.23 ± 2.6%. Statistically significant differences were observed within Massey scores and among races, with average errors that ranged from − 0.39 ± 2.3 to 0.53 ± 2.5 and − 0.55 ± 2.1 to 0.37 ± 2.7, respectively. Skin color varied widely within each self-identified race category. There was no clinically significant association between error rates and Massey-Martin scale grades and no clinically significant difference in accuracy observed between self-reported Black and White patients. In addition, self-reported race is not an appropriate surrogate for skin color.

## Introduction

Pulse oximetry is a critical monitor for assessing oxygenation status and guiding therapy in critically ill patients. Concerns regarding the impact of multiple confounding variables, including presence of nail polish, skin pigmentation, skin temperature, peripheral perfusion, motion artifact, etc., on accuracy diminished over time as the technology evolved [1–4]. A recent retrospective study renewed some of these concerns when the comparison of arterial oxygen saturation (SaO_2_) with peripheral pulse oximetry (SpO_2_) found a nearly 3-fold increase in in the incidence of occult hypoxemia in Black patients compared to White patients [5]. This report triggered a United States FDA Safety Communication emphasizing the limitations and potential inaccuracies of pulse oximetry in certain situations including home monitoring of patients with COVID-19 [6]. Subsequent studies have produced inconsistent conclusions regarding the effect of race on the accuracy of pulse oximetry in various clinical and laboratory settings [7–10]. This possible racial bias in measurement could contribute to inequities in healthcare and has triggered re-examination of the performance of additional monitoring devices. [11, 12] However, race does not uniquely characterize skin color [13]. To address these issues, we retrospectively examined SpO_2_ accuracy using data from unrelated clinical trials that also included a standardized, graded assessment of skin color via Massey-Martin scale [14]. This scale is an 11-point scale of skin pigmentation utilized to objectively scale an individual’s skin tone.

The primary hypothesis was that there was no difference in the incidence of occult hypoxemia or accuracy of pulse oximetry (defined as average error) associated with increasing Massey-Martin scale. Secondary goals included a characterization of the relationship between Massey scores and self-identified race and evaluation of the hypothesis that there was no difference in the incidence of occult hypoxemia or accuracy of pulse oximetry associated with self-identified race.

## Methods

**Fig. 1:**
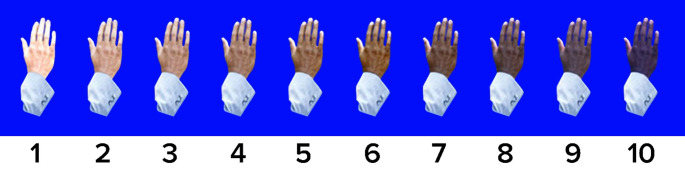
Visualization of 10 hands with example skin tones for each Massey-Martin Scale category

After Institutional Review Board review, the need for informed consent was waived due to the retrospective nature of this study and the de-identified data collected. Patients from the University of California, Davis Medical Center, Sacramento, CA were identified by their previous enrollment in contracted clinical trials that included an assessment of skin tone using the numerical Massey-Martin scale. The Massey-Martin scale, or “Massey score” is an 11-point scale, ranging from 0 being the absence of color to 10 being the darkest possible skin color [14]. This scale value was assigned by research staff at the time of study enrollment based on an illustrated guide of identical hands that differ only in skin color. (Fig. 1). A score of 0 indicates albinism, for which there were no individuals in our study, therefore it was omitted in this study.

The clinical trial data and electronic medical records (EMR) (Epic; Epic Systems, USA) accessed in 2020 & 2021 were used to collect demographic information, including self-reported race and ethnicity and all arterial blood gas (ABG) results that included SaO_2_ values with corresponding SpO_2_ values from the operating room (OR) anesthesia records or Intensive Care Unit (ICU) vital signs flowsheets throughout the patient’s hospitalizations. Both OR and ICU measured SpO_2_ with a Philips physiologic monitor (Philips Healthcare, Andover, MA, USA) and SpO_2_ module equipped with the Masimo SET^®^ technology (Masimo Inc, Irvine, CA, USA).

Corresponding SpO_2_ values were then recorded for all PaO_2_ measurements ≤ 125mmHg. These values were time matched to the closest recorded value in the EMR, with most SpO_2_ data points being less than 15 min from the associated SaO_2_ measurement. The differences between SaO_2_ and SpO_2_ values were compared as a function of both the patient’s Massey score and self-reported race. Occult hypoxemia was defined as SpO_2_ greater than 91% with SaO_2_ less than 88%, and the incidence was recorded for all patient groups.

Demographic data including self-reported race were summarized. Patients were grouped based on Massey score and self-reported race. Group comparisons were performed with the Kruskal-Wallis and Dunn’s multiple comparisons test using GraphPad Prism version 9.2.0 for Windows (GraphPad Software, San Diego, California USA, www.graphpad.com).

## Results

Massey score skin color assessments were available from 742 patients between October of 2012 and October of 2021. All patients were enrolled in one of ten contracted clinical trials sponsored by Masimo, Inc. These trials included both observational and interventional evaluations of current or developmental non-invasive monitoring technology in adult patients for elective cardiac or general surgical procedures. Massey score was a standard assessment recorded for all patients in these trials at the time of enrollment. Study enrollment logs were used to identify patients with potential data for collection and analysis. For these patients, all ABG values from throughout their hospitalization (both clinical trial and standard of care) were collected and reviewed. No SpO_2_ data was collected from any developmental, non-FDA approved devices. 579 patients had at least one PaO_2_ measurement ≤ 125mmHg and were included in this study. The average patient age (± SD) was 63.6 ± 13.9 years (minimum 18, maximum 91). In this study group, 34.5% were female and 65.5% were male. A total of 4030 individual values associated with PaO_2_ ≤ 125mmHg were available for comparison and analysis. The number of samples per patient varied from 1 to 123 with an average (± SD) of 7 ± 13.7. There was a wide range of PaO_2_ values below 125mmHg, with the lowest being 34mmHg. 139 (3.5%) samples had an PaO_2_ less than 60 mmHg. The mean ± SD PaO_2_ was 94.4 ± 18.6 mmHg. The median [95%CI] PaO_2_ value was 93 [91,93] mmHg. Corresponding SaO_2_ values ranged from 65 to 100%, with the mean ± SD being 96.3 ± 3.0% and the median [95%CI] 97 [97,97] %. For SpO_2_, there was a range of 57–100%, with a mean ± SD of 96.1 ± 3.4%, and a median [95%CI] of 97 [97,97] %. The average error (defined as SaO_2_-SpO_2_) ± SD was 0.23 ± 2.6% with a median [95%CI] of 0 [0,0].

Occult hypoxemia had an overall incidence of 0.5% (20/4030). Within this subset, PaO_2_ ranged from 48 to 115 mmHg, average ± SD 63 ± 16.9mmHg, median [95%CI] 58 [53,64] mmHg. The SaO_2_ ranged from 80 to 87%, average ± SD 85 ± 2.3%, median [95%CI] 86 [83,87] %. The SpO_2_ ranged from 92 to 99%, average ± SD 94 ± 1.6%, median [95%CI] 94 [93,94] %. The SpO_2_ - SaO_2_ difference ranged from − 5 to -12%, average ± SD -9 ± 3.0%, median [95%CI] -8 [-7, -12] %. The incidence of occult hypoxemia was not frequent enough to allow statistical evaluation as a function of either Massey score or self-identified race.

### Results based on massey score

**Fig. 2:**
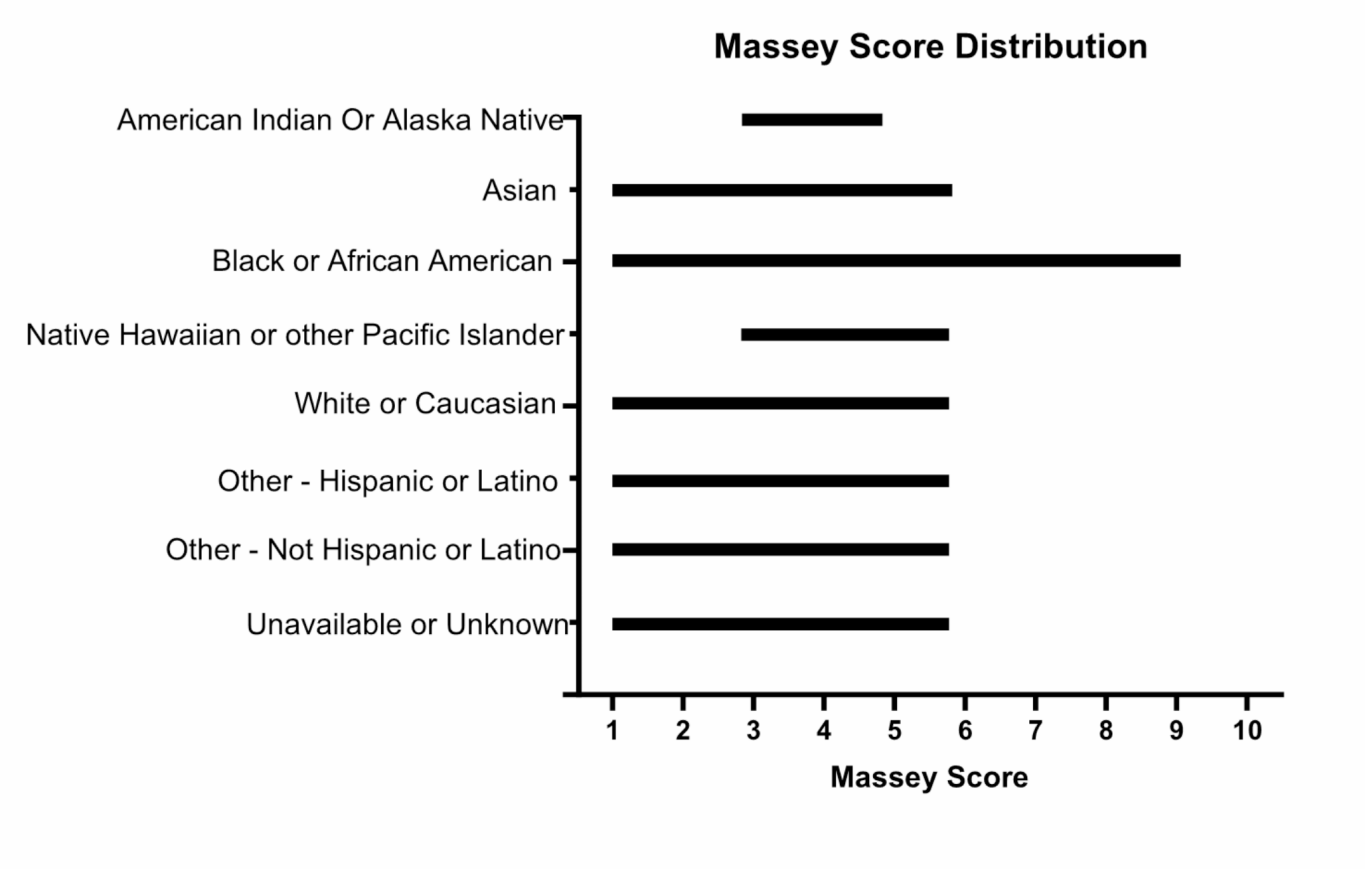
Massey Score distribution according to race category

Within each self-identified racial group there was a wide range of Massey scores (Fig. 2). Patients who self-identified as Black had Massey scores that ranged from 1 to 9 with a median score of 6 (95% CI 5,7). Patients who self-identified as White had scores that ranged from 1 to 6 with a median score of 2 (95% CI 2,3). There were no individuals in our study with Massey scores of 0 or 10.

**Fig. 3:**
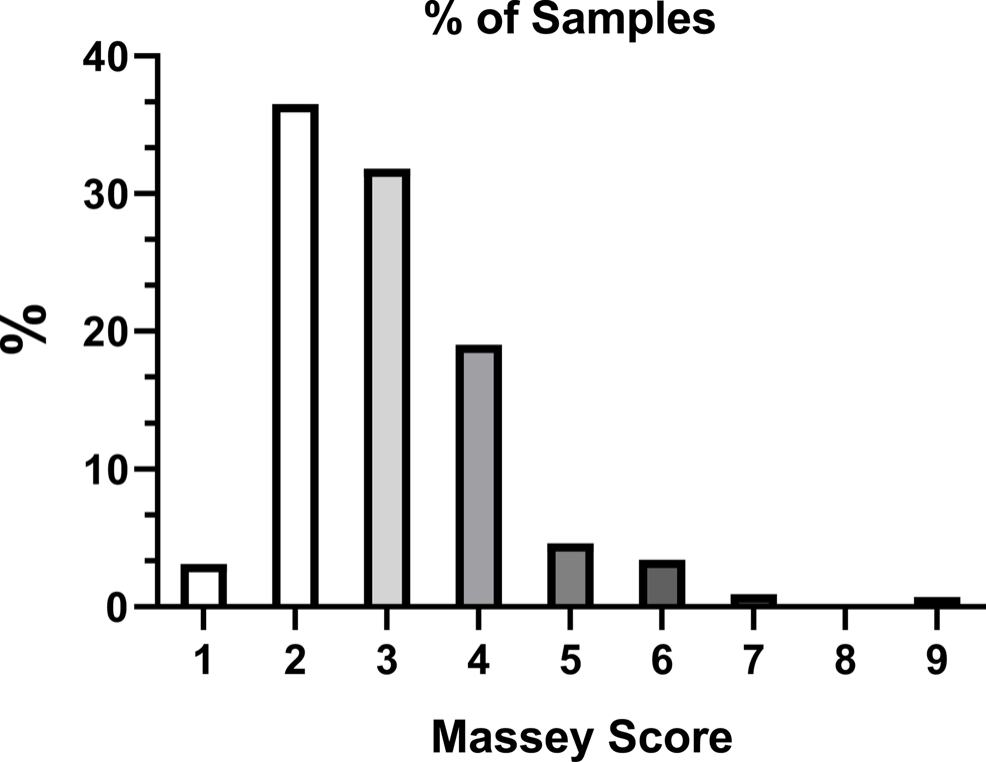
Percent of samples according to Massey Score

As summarized in Table 1, the majority of patients in this study group had Massey scores of 2 and 3. Only 4.3% of the patients in this study group had Massey scores of 6 or greater. (Fig. 3) This skewed distribution of Massey scores was associated with increased variations of error ranges, in particular, Massey score = 8. This group contained only 5 values for comparison, making up 0.12% of the 4030 total values. Massey Scores 5–8 had a negative median error, with device SpO_2_ percent saturation readings on average higher than reported SaO_2_ readings.

**Table 1:** Number of patients and values with median error and mean error according to Massey Score

Massey Score	# of patients	# of values	Median Error 95% CI [min, max]	Mean Error ± SD
1	27	123	0 [0,1]	0.53 ± 2.45
2	221	1471	0 [0,0]	0.46 ± 2.64
3	196	1281	0 [0,0]	0.38 ± 2.55
4	80	764	0 [-1,0]	-0.20 ± 2.90
5	30	184	-1 [-1,0]	-0.35 ± 2.26
6	14	137	-1 [-1,0]	-0.39 ± 2.28
7	7	38	-1 [-2,1]	0.21 ± 2.95
8	3	5	-2 [-3,-1]	-2.20 ± 0.86
9	1	27	0 [-1,1]	-0.30 ± 1.56
Total:	579	4030		

**Fig. 4:**
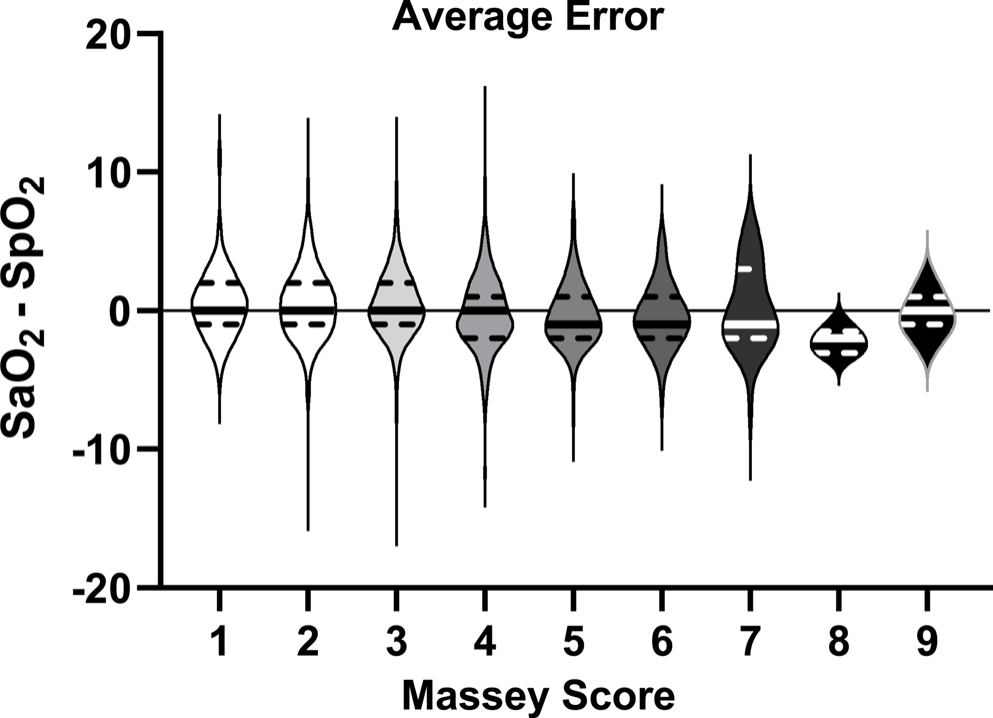
Violin plot of average errors according to Massey Score

The average errors for each Massey score group were compared and are presented as a violin plot in Fig. 4. The highest mean error was − 2.2 (SD 0.86), which correlated to category 8 on the Massey scale. The second highest mean error was for a Massey score of 1 at 0.53 (SD 2.5). Given the greater variation and small sample size, the values for a Massey score of 8 were omitted from the comparative analysis. Analysis of Variance (Kruskal-Wallis) demonstrated a statistically significant difference in the mean errors among the Massey score groups (*p* < 0.0001). Multiple comparisons revealed statistically significant differences between group 1 and group 5 (0.53 ± 2.5 vs. -0.35 ± 2.3, *p* = 0.04). Group 2 (0.46 ± 2.6) was statistically different from groups 4 (-0.20 ± 2.9, *p* < 0.0001), 5 (-0.35 ± 2.3, *p* = 0.0002) and 6 (-0.39 ± 2.3, *p* = 0.002), which were not different from each other. Group 3 (0.38 ± 2.6) was statistically different from groups 4 (-0.20 ± 2.9, *p* < 0.0001), 5 (-0.35 ± 2.3, *p* = 0.0003) and 6 (-0.39 ± 2.3, *p* = 0.003). In all cases these differences were within the expected accuracy of this monitor (-0.35 ± 2.3) [15] and not clinically significant. Similarly, there was no apparent association of error rates and skin color over the gradations of the Massey-Martin scale.

### Results based on self-identified race

As summarized in Table 2, the proportion of patients who self-identified as American Indian or Alaska Native was 0.7%, Asian was 5.0%, Black or African American was 5.7%, Native Hawaiian or other Pacific Islander was 0.7%, White was 69.9%, other- Hispanic or Latino was 10.9%, other-not Hispanic or Latino was 4.7% and unavailable or unknown was 2.1%. Since the distribution of the values was not normal, both median and mean errors are reported. The median error for all races was 0, except Asian, which had a median error of -1 (95% CI -1, -0.8). The mean error was greatest for race categories of Asian (mean ± SD [-0.51 ± 2.26]) and Black or African American (mean ± SD [-0.55 ± 2.13]). Analysis of Variance (Kruskal-Wallis) demonstrated a statistically significant difference in the mean errors among the racial groups (*p* < 0.0001). Multiple comparisons revealed statistically significant differences between the Asian and White patients (mean ± SD [0.17 ± 2.54], *p* = 0.0002), Other-Hispanic or Latino (mean ± SD [-0.01 ± 2.64], *p* = 0.017) and Unavailable (mean ± SD [0.37 ± 2.71], *p* = 0.006) patients and between the White and Other-Not Hispanic or Latino patients (*p* = 0.01). Again, these differences were within the expected accuracy of this monitor and not clinically significant.

**Table 2:** Number of patients and values, and median and mean error of pulse oximetry devices according to race as a category

Race	# of patients	# of values	Median Error 95% CI [min, max]	Mean Error ± SD
American Indian or Alaska Native	5	45	0 [-1, 0]	0.06 ± 2.45
Asian	29	134	-1 [-1, -0.8]	-0.51 ± 2.26
Black or African American	33	303	0 [0, 0]	-0.55 ± 2.13
Native Hawaiian or other Pacific Islander	5	17	0 [-2, 1]	-0.27 ± 3.78
White or Caucasian	404	2432	0 [0, 0]	0.17 ± 2.54
Other – Hispanic or Latino	63	555	0 [0, 0]	-0.01 ± 2.64
Other – Not Hispanic or Latino	27	466	0 [-1, 0]	0.50 ± 2.2
Unavailable or Unknown	12	78	0.5 [0, 1]	0.37 ± 2.71
Total:	578	4030		

## Discussion

Utilizing the Massey-Martin scale to assess the impact of skin color on the accuracy of one pulse oximetry device, we found differences in SaO_2_ to SpO_2_ were within the expected range of error of the devices and not clinically significant. There were scattered statistical differences in accuracy associated with both Massey-Martin scale and race, but no trend in the association between error rate and increasing Massey score.

A 1990 overview of respiratory function monitoring highlighted the revolutionary impact of pulse oximetry and reviewed the limitations of technology, location and potential confounding factors on the response time and accuracy of SpO_2_ measurements [2]. A contemporary clinical study evaluated the performance of pulse oximetry in ICU patients and determined that overall SpO_2_ accuracy decreased with lower SaO_2_. The error was greater in Black patients compared to White patients, but the magnitude of error did not correlate with the characterizations of skin color [1]. Alder and colleagues examined the clinical performance of pulse oximetry in the Emergency Department and found no difference in the accuracy or bias among light, intermediate, and dark skin color groups [16]. 

Potential confounding variables have been evaluated in controlled laboratory settings with smaller groups of healthy volunteers. Increased inaccuracies were observed at much lower SaO_2_ values than were reported in initial clinical studies [17]. Subsequent studies specifically evaluated the effects of skin color using two- or three-point scales [3, 18] and demonstrated statistically significant decreases in accuracy with lower saturations among different monitors and among darker skin colors, but all differences were within the expected accuracy of the technology.

The technology of oximetry devices has evolved, and performance of pulse oximeters showed improved accuracy. In 2018, Ebmeier and colleagues reported that only body temperature, oximeter model and skin color (3-point scale) produced statistically measurable but not clinically significant increases in SaO_2_-SpO_2_ differences in ICU patients [19]. In the laboratory setting, improved performance of oximeters with both patient motion and low perfusion states was reported but the impact of differences in skin color was not evaluated [4]. 

Interest in errors associated with skin color was rekindled by a retrospective study which used race as an independent variable and found the incidence of occult hypoxemia to be over 3 times higher in Black patients than in White patients [5]. This prompted the US FDA to release a Safety Communication emphasizing that providers and patients should be aware of the limitations of these devices [6]. Because pulse oximeters were so widely used during the COVID-19 pandemic to assess oxygenation status, the higher rates of occult hypoxemia in Asian, Black and Hispanic patients was especially concerning, as this could have contributed to worse outcomes for these patients [20]. 

A subsequent study of over 46,000 patients showed a higher prevalence of occult hypoxemia in non-White racial groups, but in a univariate analysis noted lack of significance in bias of SpO_2_ measurements in non-White racial groups overall. They noted that this finding could have been due to the wide range of skin-pigmentation among non-White racial groups [21]. Additional clinical studies also found occult hypoxemia to be higher in Black patients than in White patients. A retrospective examination of data from 7,693 ICU patients found higher rates of occult hypoxemia in all ethnic minority groups when compared to White patients [8]. Valbuena and colleagues retrospectively examined the incidence of occult hypoxemia in over 30,000 patients outside the ICU and found a higher incidence in both Black and Hispanic patients compared to White patients [7]. In a smaller review a higher incidence of occult hypoxemia was noted in Black vs. White patients, but no differences in Asian or Hispanic patients [22]. In contrast, a retrospective study of ICU patients showed no difference in bias or incidence of occult hypoxemia associated with race [9] and a smaller laboratory-based study also showed no significant difference in error (SaO_2_-SpO_2_) or occult hypoxemia incidence in Black vs. White subjects [10]. 

Pulse oximetry devices intended for medical use in the United States are required to undergo clinical testing for accuracy. Premarket assessment guidance issued in 2013 recommended the evaluation of performance in subjects with different skin pigmentation. Specifically, desaturation studies should include 10 or more healthy subjects that vary in age and gender, include 200 or more paired observations of SpO_2_-SaO_2_, and for the study subjects to have a range of skin pigmentation, including at least 2 darkly pigmented subjects or 15% of the study group, whichever is larger [23]. Pulse oximeters approved for use by the US Food and Drug Administration are expected to have a 2–3% root mean square accuracy of measurement at arterial blood gas saturations of 70–100%.^6^ The reported accuracy of the pulse oximetry technology used in this study exceeds this recommendation (1.4%) [10]. 

Retrospective studies of the impact of skin color on the accuracy of pulse oximetry are compromised because skin color is not a standard part of the medical record and race is not a proxy for skin color. Graded scales such as the Massey-Martin scale allow for objective measurement of this variable. The Massey score, initially used in large surveys, has been subsequently used in studies evaluating skin color and its association with America’s racial hierarchy, economic status, and discrimination and has shown excellent reliability. [24–29] Utilizing this scale rather than self-reported race offers a graduated, objective characterization of skin pigmentation.

The findings of this study are distinguished from previous retrospective data reviews by the objective characterization of skin color in contrast to the use of race as a surrogate for this variable. Race is not binary and therefore necessitates a more nuanced scale to characterize skin tones. This data set reflects diverse clinical settings, but conclusions are limited to a single institution, with a single, advanced oximetry technology. Additional limitations also require consideration. Most prominent is the skew with smaller numbers of measurements in patients with darker skin tones. 90% of the measurements are from patients with Massey scores of 1–4. Additionally, the use of a single pulse oximeter precludes extrapolation to other devices. Lastly, as a retrospective study, the time-match for the SpO_2_ and SaO_2_ values is limited. Low saturations frequently occur during periods of dynamic physiologic change that are not well captured by the time-resolution of the EMR. Future evaluations designed to address these limitations could include prospective data collections with accurately time-matched samples. Study designs should also capture a wide and balanced distribution of skin tones.

In summary, this retrospective, single institution review did not find a clinically significant difference in the accuracy of pulse oximeters with respect to skin pigmentation. Ensuring the accuracy of pulse oximeters in all patient populations is critical for equitable care, as discrepancies in measurements can perpetuate biases in healthcare delivery. These data demonstrate that skin pigmentation may be an important variable to consider, but self-reported race is not a substitute for skin color.

## Data Availability

No datasets were generated or analysed during the current study.

## Declarations

### Artwork and illustrations

All figures were created using GraphPad Prism version 9.2.0 for Windows (GraphPad Software, San Diego, California USA, www.graphpad.com).
